# Expression of cannabinoid (CB1 and CB2) and cannabinoid-related receptors (TRPV1, GPR55, and PPARα) in the synovial membrane of the horse metacarpophalangeal joint

**DOI:** 10.3389/fvets.2023.1045030

**Published:** 2023-03-03

**Authors:** Rodrigo Zamith Cunha, Augusta Zannoni, Giulia Salamanca, Margherita De Silva, Riccardo Rinnovati, Alessandro Gramenzi, Monica Forni, Roberto Chiocchetti

**Affiliations:** ^1^Department of Veterinary Medical Sciences (UNI EN ISO 9001:2008), University of Bologna, Bologna, Italy; ^2^Faculty of Veterinary Medicine, Università degli Studi di Teramo, Teramo, Italy

**Keywords:** cannabidiol, fibroblast-like synoviocytes, G protein-related receptor 55, macrophage-like synoviocytes, peroxisome proliferator-activated receptor alpha, transient receptor potential vanilloid type 1

## Abstract

**Background:**

The metacarpophalangeal joint undergoes enormous loading during locomotion and can therefore often become inflamed, potentially resulting in osteoarthritis (OA). There are studies indicating that the endocannabinoid system (ECS) modulates synovium homeostasis, and could be a promising target for OA therapy. Some cannabinoid receptors, which modulate proliferative and secretory responses in joint inflammation, have been functionally identified in human and animal synovial cells.

**Objective:**

To characterize the cellular distribution of the cannabinoid receptors 1 (CB1R) and 2 (CB2R), and the cannabinoid-related receptors transient receptor potential vanilloid type 1 (TRPV1), G protein-related receptor 55 (GPR55) and peroxisome proliferator-activated receptor alpha (PPARα) in the synovial membrane of the metacarpophalangeal joint of the horse.

**Animals:**

The dorsal synovial membranes of 14 equine metacarpophalangeal joints were collected post-mortem from an abattoir.

**Materials and methods:**

The dorsal synovial membranes of 14 equine metacarpophalangeal joints were collected post-mortem from an abattoir. The expression of the CB1R, CB2R, TRPV1, GPR55, and PPARα in synovial tissues was studied using qualitative and quantitative immunofluorescence, and quantitative real-time reverse transcriptase PCR (qRT-PCR). Macrophage-like (MLS) and fibroblast-like (FLS) synoviocytes were identified by means of antibodies directed against IBA1 and vimentin, respectively.

**Results:**

Both the mRNA and protein expression of the CB2R, TRPV1, GPR55, and PPARα were found in the synoviocytes and blood vessels of the metacarpophalangeal joints. The synoviocytes expressed the mRNA and protein of the CB1R in some of the horses investigated, but not in all.

**Conclusions and clinical importance:**

Given the expression of the CB1R, CB2R, TRPV1, GPR55, and PPARα in the synovial elements of the metacarpophalangeal joint, these findings encouraged the development of new studies supporting the use of molecules acting on these receptors to reduce the inflammation during joint inflammation in the horse.

## Introduction

The metacarpophalangeal joint is a high mobility structure which undergoes enormous loading during locomotion and jumping in the horse (1), so much so that it is the most commonly reported joint affected by traumatic and degenerative lesions in equine athletes (2) and results in lameness in thoroughbred racehorses (3, 4). Currently, there is no specific cure for joint disease, and the multimodal pharmacological treatment does not act on the cause of joint inflammation but is aimed at slowing its progression, minimizing/reducing pain, and increasing function and performance (5). In recent decades, some molecules have given encouraging results for the treatment of osteoarthritis, even if it is difficult to draw definitive conclusions (6). Therefore, there is a need for improving the understanding of the pathophysiology and mechanisms of joint pain in order to develop safe and effective drugs to alleviate symptoms in horses with synovitis and osteoarthritis (OA) (7). Joint inflammation can affect cartilage, bone and the synovial membrane within the joint (8). However, regardless of which intra-articular tissue type is first affected, the synovial membrane seems to modulate and reinforce the inflammatory responses of the joints (9, 10). Hence, the synovial membrane is key to enhancing the understanding of the pathophysiological processes within the synovial joint.

The wall of the joint capsule is composed of two distinct layers: the external and thick fibrous layer (*stratum fibrosum*), and the inner and thin synovial membrane (*synovium*). The cells of the intimal lining of the *synovium* secrete the fluid into the joint cavity (synovial fluid), remove debris and are involved in the production of cytokines/molecules which may modulate the joint inflammation (9–13). Two types of synoviocytes lining the luminal side of the joint capsule have been described in depth in humans and animals (14, 15), including horses (11, 13, 16, 17): (1) macrophage-like synoviocytes (MLS), also known as type A synoviocytes, and (2) fibroblast-like synoviocytes (FLS), also known as type B synoviocytes. Embedded in a thin layer of connective tissue rich in fenestrated capillaries, the synoviocytes produce and control the synovial fluid. Fibroblast-like synoviocytes, the dominating cell-type in the synovial intima, produce hyaluronic acid and other lubricating synovial additives of the synovial fluid, and also matrix components (such as collagens, proteoglycans and laminin) and degrading enzymes (such as matrix metalloproteinases [MMPs] and other proteases) (18). In cultured human FLS, it has been shown that these cells organize a basement membrane-like extracellular matrix, capable of supporting monocyte survival and compaction into the lining (19). A more recent study has shown that fibroblasts might also provide anchorage to the MLS and are also a source of key survival factors of the MLS (20).

Although FLS morphologically differ from the other fibroblasts, these cells may express the typical fibroblast markers vimentin (21) or, uniquely in the horse, the neuronal marker Protein Gene Product 9.5. (16). However, due to the specific functions of the FLS in the synovial lining (22), there are only a few reports of selective markers of FLS, differentiating them from other musculoskeletal fibroblasts (13).

Macrophage-like synoviocytes are macrophages not derived from bone-marrow immune cells (monocytes) but derived from cells which disperse into the tissues during embryonic development and are resident in the joint (23). Macrophage-like synoviocytes may be distributed unevenly adjacent to the joint lumen (11) or, as has recently been described in mice, may congregate to form an internal immunological barrier at the synovial lining which physically seclude the joint (24, 25). General resident macrophage-markers, such as CD11b, CD14, CD68, and CD206, may be expressed by horse MLS (26).

The strong need to develop a treatment for synovial inflammation, cartilage degeneration, and bone deformation has led to research regarding the involvement of the immunomodulatory endocannabinoid system (ECS) in the development of OA (12, 27–32). The involvement of the ECS in immunocytes and macrophages, as well as in regulatory actions on sensory nociceptors to ameliorate pain in OA, has been described (33). The ECS consists of endocannabinoid molecules involved in signaling processes, along with G-protein-coupled receptors (GPCRs) and enzymes associated with ligand biosynthesis, activation and degradation (33). Endocannabinoids and endocannabinoid-like lipid mediators, such as palmitoylethanolamide (PEA) (34), the phytocannabinoids derived from *Cannabis sativa*, such as Δ-9-tetrahydrocannabinol (THC), cannabidiol (CBD), cannabigerol, cannabichromene, and cannabinol (35, 36), and the synthetic cannabinoids all act on canonical cannabinoid-1 (CB1R) and−2 (CB2R) receptors. They also act on cannabinoid-related receptors, such as the transient receptor potential (TRP) channels, the G protein-coupled receptors (GPCRs), the nuclear peroxisome proliferator-activated receptors (PPARs), and the serotonin receptors (12, 35, 37–39).

There are studies showing that the activation of CB1R and CB2R, which are expressed in human, mouse, and horse synoviocytes (28, 40–44), can induce potent anti-inflammatory effects and modulate arthritic disease (31, 43, 45). The TRP vanilloid 1 (TRPV1) ion channel, which is expressed in human and rat synoviocytes (41, 46), might also be a possible target for treating joint diseases (46).

There are no studies which have reported the expression of G protein-coupled receptor 55 (GPR55) or PPARα in synoviocytes. However, GPR55 has been localized in human chondrocytes, osteoclasts and osteoblasts (47, 48) and there are studies indicating that PPARα agonists may exert beneficial effects on OA due to their anti-inflammatory effects (49, 50).

Given the aforementioned data obtained in other species, it is conceivable that the receptors of the endocannabinoid system could be expressed in the horse synovial membrane and represent a pharmacological target for the treatment of joint diseases. Currently, only a few reports have been published regarding the cannabinoid and cannabinoid-related receptors of the horse joint *synovium* (4, 44).

Thus, the current study was designed to identify the mRNA of *Cnr1, Cnr2, TRPV1, GPR55*, and *PPARA* and to immunohistochemically localize these receptors in the synovial membrane of the equine metacarpophalangeal joint.

## Materials and methods

### Animals

The metacarpophalangeal joints of 14 healthy horses (9 females and 5 males), ranging from 2 to 20 years of age (mean: 12 years; SD ± 6.5), which were slaughtered for consumption were collected from the thoracic limbs post-mortem. The breeds included 1 Avelignese, 1 Italian thoroughbred, and 12 half-breeds. The distal forelimbs were removed at the carpal joint to obtain the metacarpophalangeal joints.

A complete cell blood count (CBC) and routine serum biochemical analyses were carried out using blood samples taken at the time of exsanguination. The horses, which did not show lameness of either the thoracic or the pelvic limbs, were considered to be healthy on the basis of a summary clinical visit prior to slaughter, normal results of the CBC count and routine serum biochemical analyses. In addition, the presence of OA or other pathological conditions were excluded by post-mortem gross and histological evaluation.

According to Directive 2010/63/EU of the European Parliament and of the Council of 22 September 2010 regarding the protection of animals used for scientific purposes, Italian legislation (D. Lgs. n. 26/2014) does not require any approval by competent authorities or ethics committees as this study did not influence any therapeutic decisions.

### RNA isolation and reverse transcription

Total RNA extraction was performed using TRI Reagent (Molecular Research Center In, Cincinnati, OH, USA) and a NucleoSpin RNA II kit (Macherey-Nagel GmbH & Co. KG, Düren, Germany) according to the manufacturer's instructions. Dorsal synovial membranes, collected from eight horses, were homogenized in TRI Reagent (50 mg/ml) with IKA T10 Basic Ultra-Turrax; 200 μL of chloroform were subsequently added to the suspension which was then mixed well. After incubation at room temperature (RT) (10 min), the samples were centrifuged (12,000 × g for 10 min) and the aqueous phase recovered. An equal volume of 70% ethanol was added, and the RNA containing phase was applied to the NucleoSpin RNA Column and cleaned in the further steps of the protocol. Finally, 60 μl of molecular biology water was applied into the column membrane, centrifuged, and RNA was eluted into a new Eppendorf-type tube. After nanospectrophotometric quantification (DeNovix, DeNovix Inc. Wilmington, DE USA), the total RNA (500 ng) was reverse transcribed to cDNA using 5X iScript RT Supermix (Bio-Rad Laboratories Inc., Hercules, CA, USA) in a final volume of 20 μl.

### Quantitative real-time PCR (RT-PCR) gene expression analysis

To evaluate gene expression profiles, quantitative real-time PCR (qPCR) was carried out in a CFX96 thermal cycler (Bio-Rad Laboratories Inc.) using SYBR green detection (Cat.172-5121, Bio-Rad laboratories Inc) for target genes. Specific primers for the horse were designed (Beacon Designer 2.07, Premier Biosoft International, Palo Alto, CA, USA) using the target genes for *Cnr1* (Cannabinoid receptor 1), *Cnr2* (Cannabinoid receptor 2), *GPR55, PPARA*, and *TRPV1* (Table 1). Regarding the reference genes, *GAPDH* (Glyceraldehyde-3-phosphate dehydrogenase), *HPRT* (Hypoxanthine phosphoribosyltransferase 1) and *ACTB* (Actin B) were selected on horse sequences as previously reported (51). All the amplification reactions were carried out in 20 μl and analyzed in duplicate; the reaction contained 10 μl of iTaq Universal SYBR Green Supermix (Cat.172-5121, Bio-Rad laboratories Inc.), 0.8 μl of the forward and reverse primers (5 μM each) of each target gene, 1.5 μl cDNA, and 7.7 μl of water. The real-time procedure included an initial denaturation period of 3 min at 95°C, 40 cycles at 95°C for 15 s, and 60°C for 30 s, followed by a melting step with ramping from 55 to 95°C at a rate of 0.5°C/10 s. To validate the primers chosen, extraction and qPCR from a positive control (equine amygdala) were also performed. The specificity of the amplified PCR products was confirmed by agarose gel electrophoresis and melting curve analysis. The relative expressions of the interest genes (IG) were normalized based on the geometric mean of the three reference genes (RG) (52). The relative mRNA expression of the genes tested was evaluated as using the ΔCt method with ΔCt = (Ct geometric mean RG – Ct IG), which directly correlated with the expression level.

**Table 1:** Primer sequence used for quantitative real time PCR analysis.

**Gene**		**Primer sequence (5^′^->3^′^)**	**PCR size (bp)**	**Accession number**	**References**
Cnr1	F	AACCTACCTGATGTTCTGGATTGG	147	NM_001257151.1	Present study
	R	GATGTGTGGATGATGATGCTCTTC			
Cnr2	F	CTCCTGTTCATTGCCATCCTCTTCTCTG	114	NM_001257179.1	Present study
	R	CTGCCTGTCTTGGTCCTGGTGTTC			
GPR55	F	CCGCCTTCTCCTCCTTCCTCTCAG	118	XM_023642534.1	Present study
	R	TCACTCCTCCACACCCATTTCTACCC			
PPARA	F	CATTGGCGAGGACAGTTGCGGAAG	182	NM_001242553.1	Present study
	R	CGATGTTCAATGCTGTGCTGGAAGATTC			
TRPV1	F	ACCTGTGTCGCTTCATGTTTGTCTACC	105	XM_014727972.2	Present study
	R	ATTCAGCCAGCACGGAGTCATTCTTC			

### Immunofluorescence

Dorsal synovial membrane specimens (~2 cm × 1 cm) were dissected with a scalpel and fixed for 48 hours at 4°C in 4% paraformaldehyde in phosphate buffer (0.1 M, pH 7.2), subsequently rinsed in phosphate-buffered saline (PBS; 0.15 M NaCl in 0.01 M sodium phosphate buffer, pH 7.2) and stored at 4°C in PBS containing 30% sucrose and sodium azide (0.1%). The following day, the tissues were transferred to a mixture of PBS−30% sucrose–azide and Optimal Cutting Temperature (OCT) compound (94-4583, Sakura Finetek Europe, Alphen aan den Rijn, The Netherlands) at a ratio of 1:1 for an additional 24 h before being embedded in 100% OCT in Cryomold^®^ (94-4566, Sakura Finetek Europe). The samples were prepared by freezing the tissues in isopentane cooled in liquid nitrogen. Cryosections (14 μm thick) of synovial membrane were cut on a cryostat (MC5000, Histo-Line Laboratories, Pantigliate, Italy), and mounted on polylysinated slides (HL26765, Histo-Line Laboratories).

The cryosections were hydrated in PBS and processed for immunostaining. To block non-specific bindings, the sections were incubated in a solution containing 20% normal donkey serum (Colorado Serum Co., Denver, CO, USA), 0.5% Triton X- 100 (Sigma Aldrich, Milan, Italy, Europe), and bovine serum albumin—BSA (1%) in PBS for 1 hour at RT (22–25°C). The cryosections were incubated in a humid chamber overnight at RT with the anti-CB1R, -CB2R, -TRPV1, -GPR55, and PPARα antibodies (single immunostaining) or with a cocktail of primary antibodies (double immunostaining) (Table 2) diluted in 1.8% NaCl in 0.01 M PBS containing 0.1% sodium azide. After washing in PBS (3 × 10 min), the sections were incubated for 1 h at RT in a humid chamber with the secondary antibodies (Table 3) diluted in PBS. The cryosections were then washed in PBS (3 × 10 min) and mounted in buffered glycerol at pH 8.6 with 4′,6-diamidino-2-phenylindole–DAPI (Santa Cruz Biotechnology, Santa Cruz, CA, USA). To identify macrophages and fibroblasts, the anti-ionized calcium binding adapter molecule 1 (IBA1) (53) and the anti-vimentin (Clone V9) (42) antibodies were used, respectively.

**Table 2:** Primary antibodies used in the study.

**Primary antibody**	**Host**	**Code**	**Dilution**	**Source**
CB1R	Rabbit	ab23703	1:100	Abcam
CB2R	Mouse	sc-293188	1:50	Santa Cruz
CB2R	Rabbit	PA1-744	1:250	Thermo Fisher
GPR55	Rabbit	NB110-55498	1:200	Novus Biol.
IBA1	Goat	NB100-1028	1:80	Novus Biol.
PPARα	Rabbit	NB600-636	1:200	Novus Biol.
TRPV1	Rabbit	ACC-030	1:200	Alomone
Vimentin	Mouse	IS630 (Clone V9)	1:600	Dako

*Primary antibody suppliers: Abcam, Cambridge, UK; Alomone, Jerusalem, Israel; Dako Cytomation, Golstrup, Denmark; Novus Biologicals, Littleton, CO, USA; Santa Cruz Biotechnology, California, USA; Thermo Fisher Scientific, Waltham, MA USA*.

**Table 3:** Secondary antibodies used in the study.

**Secondary antibody**	**Host**	**Code**	**Dilution**	**Source**
Anti-mouse IgG Alexa-594	Donkey	A-21203	1:500	Thermo Fisher
Anti-goat 594	Donkey	ab150132	1:600	Abcam
Anti-rabbit 488	Donkey	A-21206	1:1000	Thermo FISHER

*Secondary antibody suppliers: Abcam, Cambridge, UK; Thermo Fisher Scientific, Waltham, MA USA*.

### Specificity of the primary antibodies

#### Antibodies anti-cannabinoid receptors

The rabbit anti-CB1R antibody utilized in the present study had already been tested using Western blot (WB) analysis on horse tissues (54).

The rabbit anti-CB2R antibody (PA1-744) utilized in the present study had already been tested with Western blot (WB) analysis on horse tissues (55). In the current study, another anti-CB2R antibody, raised in mice (sc-293188), was used, the specificity of which has not yet been tested on horse tissues; however, both the mouse and rabbit anti-CB2R antibodies were tested using a double-staining protocol and were co-localized in horse tissues (Supplementary Figure 1).

#### Antibodies anti-cannabinoid-related receptors TRPV1, GPR55, and PPARalpha

The specificity of the anti-TRPV1 antibody had been tested by the research group using Western blot analysis on horse tissue (53). In addition, the specificity of the anti-TRPV1 antibody had previously been tested using WB analysis on rat tissues (56).

The immunogen used to obtain the anti-GPR55 antibody was the synthetic 20 amino acid peptide from the third cytoplasmic domain of Human GPR55 in amino acids 200–250. The homology between the full amino acid sequences of the horse and human GPR55 was 80%, and the correspondence with the specific sequence of the immunogen was 78%. This antibody, which has recently been used in horse sensory neurons (53), had previously been tested on rat and dog dorsal root ganglia (DRG) using immunofluorescence (57) and on mice tissues using WB analysis (58). However, the WB analysis had not been carried out on horse tissue.

The specificity of the primary anti-PPARα antibody had been tested using WB analysis on horse tissue (54). In addition, the antibody utilized had also recently been tested on rat tissue (57) as the anti- PPARα antibody reacts with rat tissue, as stated by the antibody supplier. The same anti-PPARα antibody has recently been used in horse tissues (53, 59).

#### Marker for macrophages (MLS) and fibroblasts (FLS)

The goat anti-IBA1 antibody, recently used on horse tissue (53), was directed against a peptide having the sequence C-TGPPAKKAISELP, from the C Terminus of the porcine IBA1 sequence. Horse and porcine IBA1 molecules share 92.3% identity (https://www.uniprot.org/), and it is plausible that the antibody used can also recognize IBA1 in the horse.

The mouse anti-vimentin antibody (Clone V9) had already been used to label fibroblasts in the horse skin (60).

### Specificity of the secondary antibody

The specificity of the secondary antibodies was tested by applying them on the sections after omitting the primary antibodies. No stained cells were detected after omitting the primary antibodies.

### Quantitative analysis

Quantitative analysis of the intensity of the expression of cannabinoid and cannabinoid-related receptors in the synovial intimal layer was carried out on 12 horses.

For each animal, and each receptor, two randomly selected images of the synovial membrane (50 μm-thick and 100 μm-wide; 5,000 μm^2^ area) were acquired (high magnification, ×400), using the same exposure time for all the images. The 50 μm-thick of synovial membrane encompassed the intimal synoviocytes and a minimal amount of underlying subintimal blood vessels, infiltrating cells and fibroblasts (61). In each image the signal intensity was analyzed using ImageJ software (Image J, version 1.52t, National Institutes of Health, Bethesda, MD, USA) (62) by standardized thresholds for brightness and contrast were determined empirically and applied to all images. The signal intensity was finally obtained using the Color histogram (gMEAN) tool of the software.

### Statistical methods

For each receptor the mean of the two values/case of signal intensity in the 12 horses were evaluated and compared. Statistical analysis was carried out using GraphPad Prism software (version 8.3, La Jolla, CA). The normality distribution of the data was assessed using the Shapiro-Wilk test. Comparisons between groups were performed with one way ANOVA Tukey's multiple comparisons test. A *P*-value ≤ 0.05 was considered significant.

### Fluorescence microscopy

The preparations were examined, by the same observer on a Nikon Eclipse Ni microscope (Nikon Instruments Europe BV, Amsterdam, The Netherlands, Europe) equipped with the appropriate filter cubes. The images were recorded with a DS-Qi1Nc digital camera and NIS Elements software BR 4.20.01 (Mountain View, Ottawa, ON, Canada). Slight contrast and brightness adjustments were made using Corel Photograph Paint whereas the figure panels were prepared using Corel Draw (Mountain View).

## Results

### qPCR for Cnr1, Cnr2, GPR55, PPARA and TRPV1

**Figure 1:**
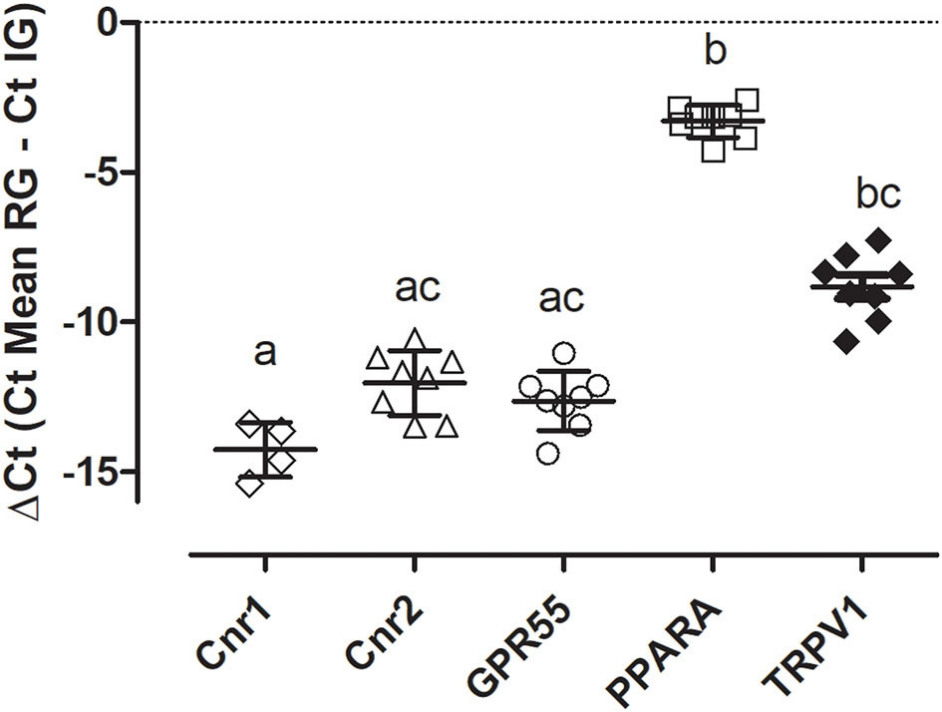
Gene expression of *Cnr1, Cnr2, GPR55, PPARA*, and *TRPV1* in equine sinovial membranes. The results are presented as ΔCt = (Ct Mean RG – Ct IG). Symbols indicate individual animals. For each gene, mean ± SD are indicated by horizontal bars. Different letters indicate statistically significant differences (*p* < 0.05, Kruskal–Wallis test, Dunn's Multiple Comparison *post-hoc* test).

Quantitative PCR data demonstrated that *Cnr2, GPR55, PPARA*, and *TRPV1* were detected in all the equine synovial samples (*n* = 8) while the transcript for *Cnr1* was detectable in only four synovial samples (50%). As reported in Figure 1, the level of gene expression was different in the synovial samples having a greater expression of *PPARA*.

### Immunofluorescence

It is necessary to point out that the identification of the two types of synoviocytes of the equine metacarpophalangeal joints is by no means a simple matter. In fact, there are some articles which testify to the fact that the horse FLS can be very similar to the MLS, from a morphological point of view (11, 16, 17). For this reason, two markers which should be selective for fibroblasts (vimentin) and for macrophages (IBA1) were used.

### Vimentin and IBA1 distribution and expression analysis

**Figure 2:**
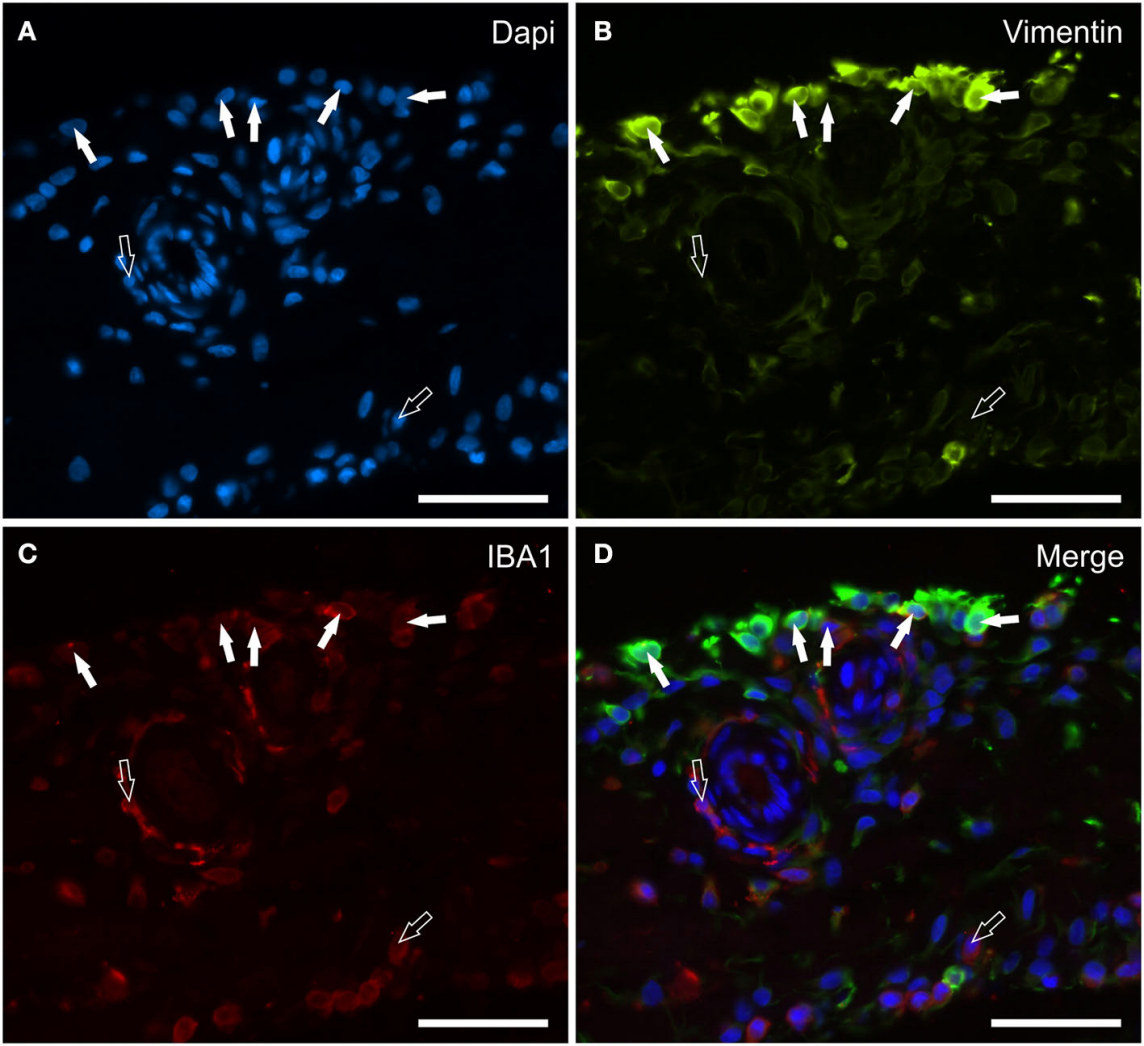
Photomicrographs of the cryosections of the synovial membrane of a horse metacarpophalangeal joint showing vimentin **(B)** and IBA1 **(C)** immunoreactivity. The white arrows indicate the DAPI (Blue) labeled nuclei **(A)** of some macrophage-like synoviocytes lining the synovial intima which co-expressed moderate IBA1 (Red) and bright vimentin (Green) immunoreactivity. The open arrows indicate subintimal macrophages, which were IBA1 immunoreactive and vimentin negative. **(D)** Merged image (Orange). Scale bar = 50 μm.

Vimentin appears to be an excellent marker for identifying the morphology of the cells lining the synovial membrane of the horse joint. Bright vimentin immunoreactivity (vimentin-IR) was mainly expressed by FLS, which were recognizable owing to their long and thin processes extending toward the joint cavity. In some portions of the synovial intima, these processes exhibited a densely arranged plexus on the surface. Co-localization studies have indicated that a proportion of moderate-to-bright vimentin immunoreactive cells (MLS) also expressed IBA1-IR (Figures 2A–D). In the subintima, the IBA1 immunoreactive macrophages showed faint or moderate vimentin-IR.

### CB1R distribution and expression analysis

**Figure 3:**
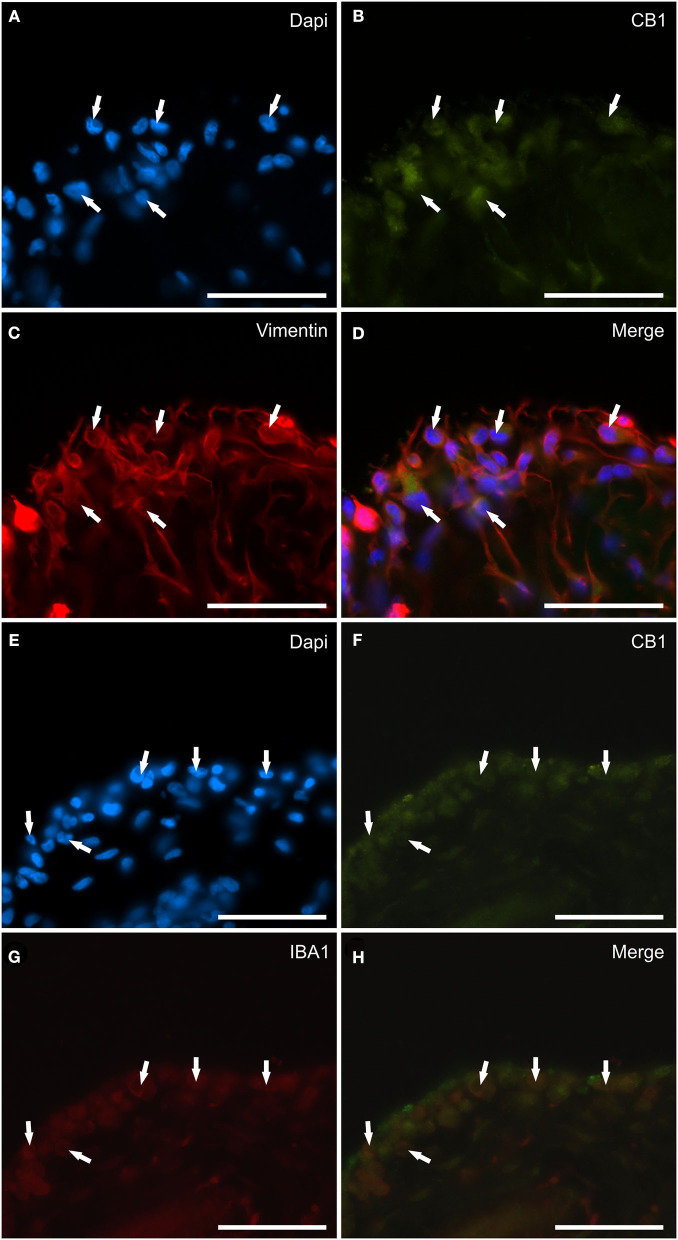
Photomicrographs of the cryosections of the synovial membrane of a horse metacarpophalangeal joint showing cannabinoid receptor type 1 (CB1) immunoreactivity in synoviocytes. **(A–D)** The arrows indicate the DAPI (Blue) labeled nuclei **(A)** of cells resembling fibroblast-like synoviocytes co-expressing faint CB1 (Green) receptor immunoreactivity **(B)** and bright vimentin (Red) **(C)** immunoreactivity. **(D)** Merged image (Orange). **(E–H)** The arrows indicate the DAPI (Blue) labeled nuclei **(E)** of round macrophage-like synoviocytes co-expressing faint CB1 (Green) receptor **(F)** and IBA1 (Red) **(G)** immunoreactivity. **(H)** Merged image (Orange). Scale bar = 50 μm.

Faint CB1R-IR was expressed by the cytoplasm of the synoviocytes; however, the CB1R-IR was detectable in only 10/14 (71 %) horses. Co-localization studies have indicated that vimentin immunoreactive FLS (Figures 3A–D) and IBA1 immunoreactive MLS (Figures 3E–H) expressed CB1R-IR. Cannabinoid receptor 1 was not expressed by blood vessels and fibroblasts.

### CB2R distribution and expression analysis

**Figure 4:**
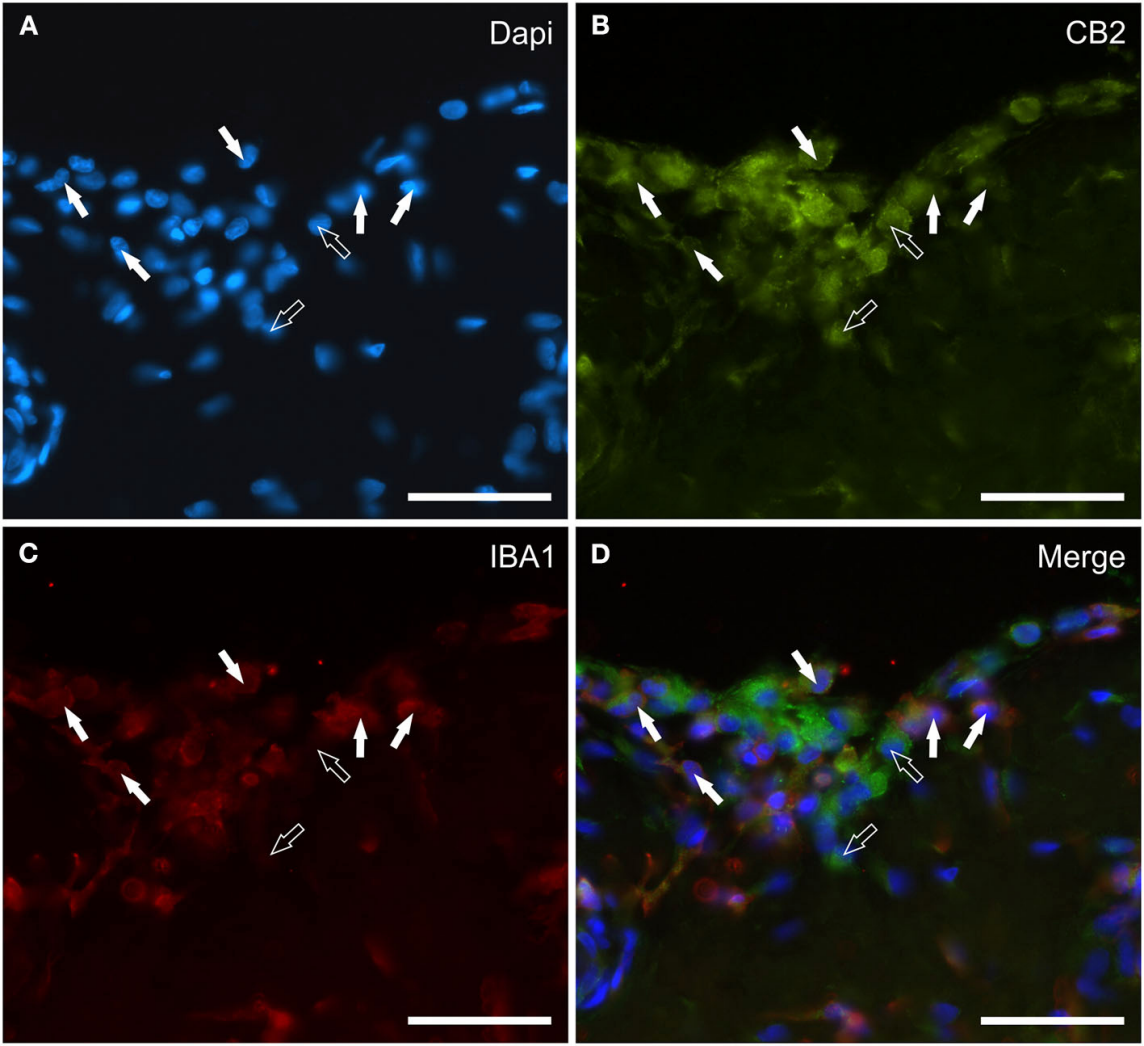
Photomicrographs of the cryosections of the synovial membrane of a horse metacarpophalangeal joint showing cannabinoid receptor type 2 (CB2) **(B)** and IBA1 **(C)** immunoreactivity. The white arrows indicate the DAPI (Blue) labeled nuclei **(A)** of some round macrophage-like synoviocytes lining the joint cavity which co-expressed IBA1 (Red) and bright CB2 (Green) receptor immunoreactivity. The open arrows indicate two cells expressing CB2 receptor immunoreactivity (likely fibroblast-like synoviocytes) which were IBA1 negative. **(D)** Merged image (Orange). Scale bar = 50 μm.

Cannabinoid receptor 2 immunoreactivity was expressed by synoviocytes, blood vessels and fibroblasts. Bright cytoplasmic CB2R-IR was observed in oval and elongated FLS and in round-shaped IBA1 immunoreactive MLS (Figures 4A–D). Co-localization with the anti-CB1R antibody showed that synoviocytes expressed both receptors in those horses in which CB1R-IR was detectable (Supplementary Figure 2). The vascular endothelial and smooth muscle cells showed bright and moderate CB2R-IR, respectively (Data not shown). Moderate CB2R-IR was also expressed by cells, likely fibroblasts, distributed in the sublining layer.

### TRPV1 distribution and expression analysis

**Figure 5:**
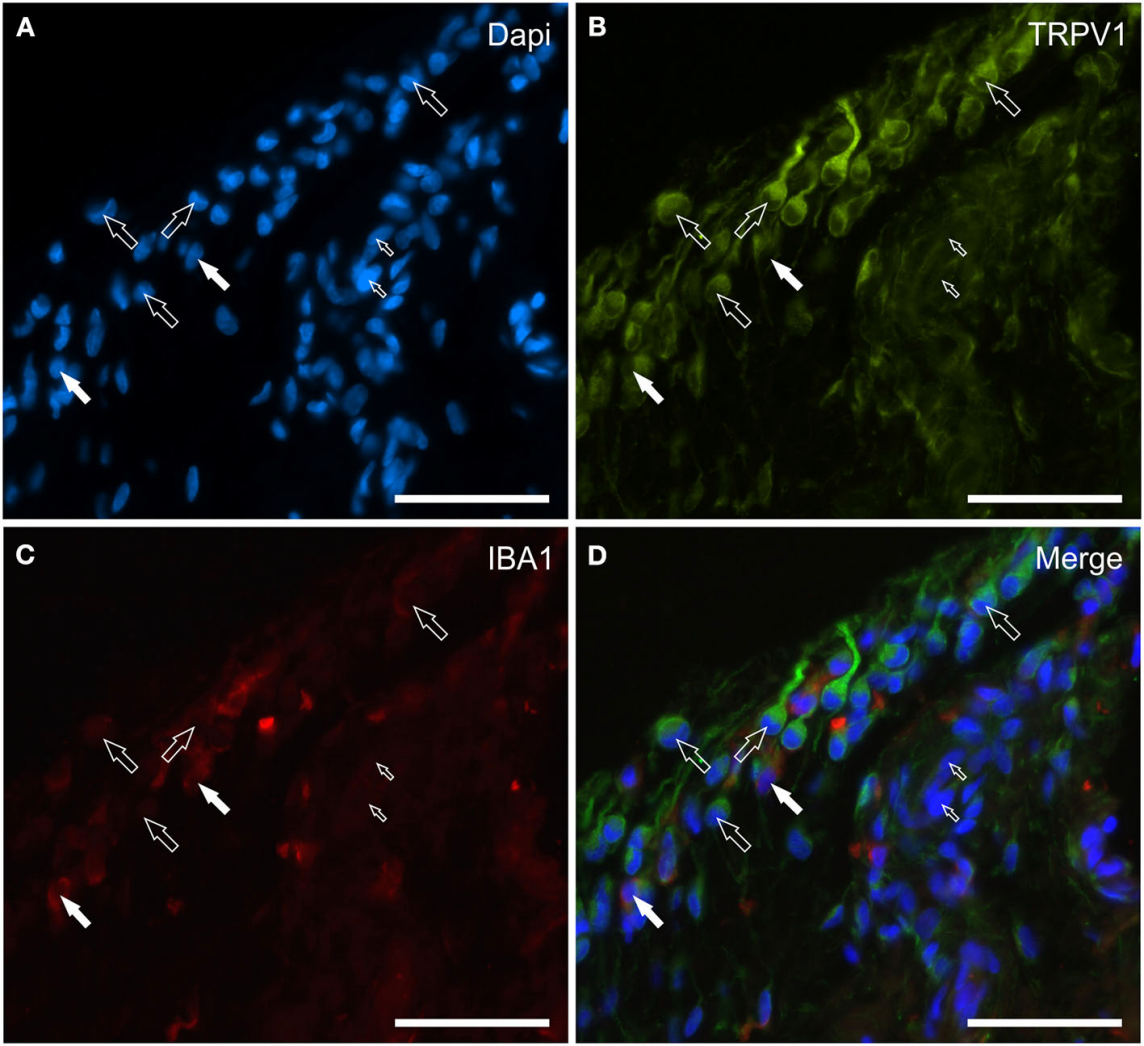
Photomicrographs of the cryosections of the synovial membrane of a horse metacarpophalangeal joint showing transient receptor potential vanilloid 1 (TRPV1) **(B)** and IBA1 **(C)** immunoreactivity. Both of the two cell types lining the synovial intima, i.e., the fibroblast-like synoviocytes (FLS) and the macrophage-like synoviocytes (MLS), showed bright TRPV1 (Green) immunoreactivity. The TRPV1 immunolabeling was also evident in the elongated cellular process of the FLS extending through the joint cavity. The white arrows indicate the DAPI (Blue) labeled nuclei **(A)** of two IBA1 (Red) immunoreactive MLS co-expressing bright TRPV1 **(B)** and moderate IBA1 **(C)** immunoreactivity. The open arrows indicate some round or elongated FLS which were TRPV1 immunoreactive and IBA1 negative. The small open arrows indicate the DAPI labeled nuclei of the endothelial cells showing moderate TRPV1 immunoreactivity. **(D)** Merged image (Orange). Scale bar = 50 μm.

**Figure 6:**
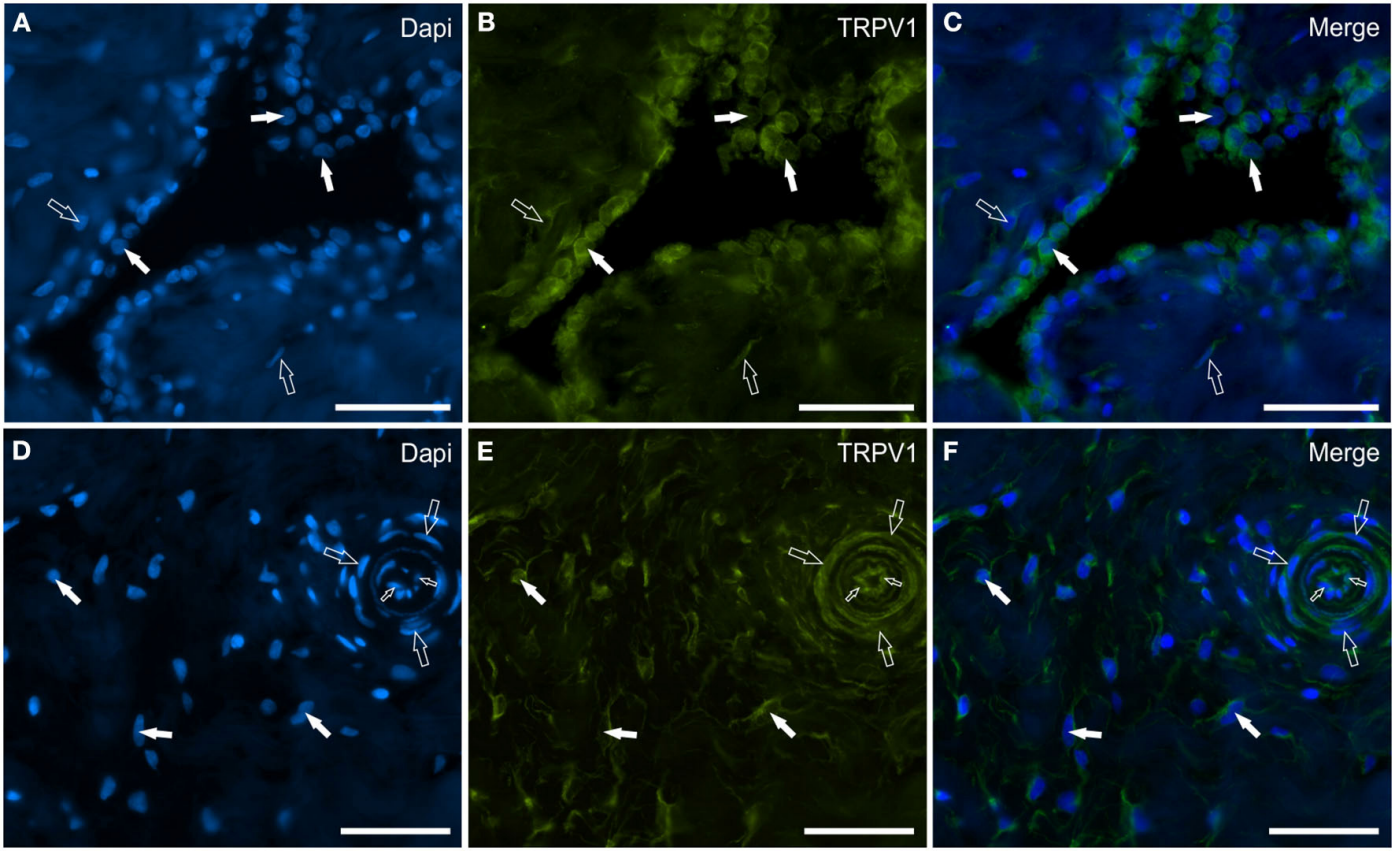
Photomicrographs of the cryosections of the synovial membrane of a horse metacarpophalangeal joint showing transient receptor potential vanilloid 1 (TRPV1) **(B, E)**, immunoreactivity in synoviocytes **(A–C)**, and fibroblast and vascular cells **(D–F)**. **(A–C**) The white arrows indicate the DAPI (Blue) labeled nuclei of some round synoviocytes expressing bright TRPV1 (Green) immunoreactivity. The open arrows indicate subintimal cells (likely fibroblasts) showing faint-to-moderate TRPV1 immunoreactivity. **(D–F)** The open arrows indicate the DAPI (Blue) labeled nuclei of some cells of the interstitial connective tissues of the synovial membrane (close to the subintima) expressing moderate-to-bright TRPV1 (Green) immunoreactivity. The open arrows and the small open arrows indicate the DAPI labeled nuclei of the vascular smooth muscle cells and endothelial cells, respectively, expressing moderate TRPV1 immunoreactivity. **(C, F)** Merged images. Scale bar = 50 μm.

Transient receptor potential vanilloid 1 immunoreactivity was expressed by synoviocytes, blood vessels and fibroblasts. Bright TRPV1-IR was mainly expressed by the cell membrane and cytoplasm of FLS and, in particular, also by their long “dendritic” processes which extended irregularly toward the luminal surface of the synovial membrane (Figures 5A–D). Co-localization between TRPV1 and IBA1 showed that TRPV1-IR was also expressed by the cell membrane and cytoplasm of MLS (Figures 5A–D). In some portions of the synovial intima, oval-shaped synovial cells, expressing moderate-to-bright TRPV1-IR, appeared to be the prevalent cells, and were aligned and organized in such a way as to form an epithelium-like monolayer with the appearance of a barrier, resembling the cellular organization recently described in the rat synovial membrane (24, 25) (Figures 6A–C). In other portions of the membrane, however, the “elongated” FLS seemed to prevail in the most superficial layer (Figures 5A–D). The endothelial cells of the capillaries adjacent to the joint lumen and arteries of the *stratum fibrosum* showed moderate cytoplasmic TRPV1-IR. The vascular smooth muscle cells also showed moderate TRPV1-IR (Figures 6D–F).

### GPR55 distribution and expression analysis

**Figure 7:**
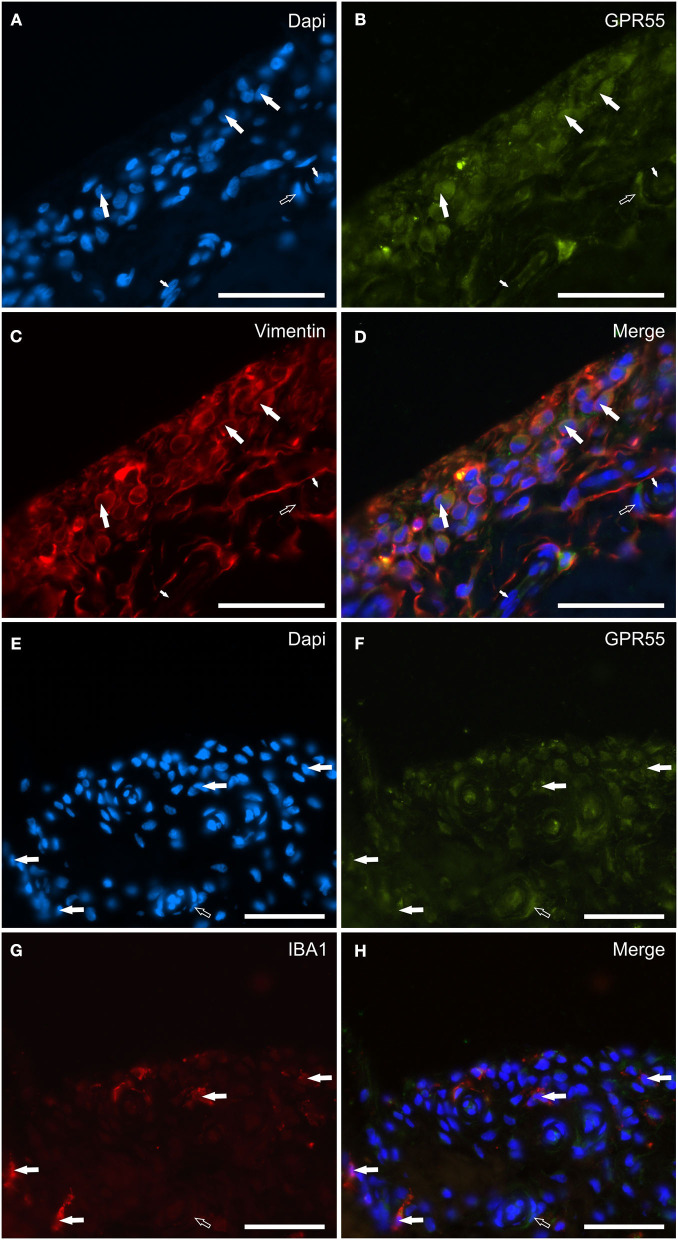
Photomicrographs of the cryosections of the synovial membrane of a horse metacarpophalangeal joint showing G protein-related receptor 55 (GPR55) immunoreactivity in synoviocytes and vascular cells. **(A–D)** The white arrows indicate the DAPI (Blue) labeled nuclei **(A)** of elongated cells resembling fibroblast-like synoviocytes co-expressing moderate GPR55 (Green) **(B)** and bright vimentin (Red) **(C)** immunoreactivity. The small white arrows indicate the DAPI labeled nuclei of the endothelial cells of the subintima blood vessels showing moderate GPR55 immunoreactivity which was also expressed by the vascular smooth muscle cells (open arrow). **(E–H)** The white arrows indicate the DAPI (Blue) labeled nuclei of macrophage-like synoviocytes co-expressing faint-to-moderate GPR55 (Green) **(F)** and bright IBA1 (Red) immunoreactivity **(G)**. The open arrow indicates the nuclei of a vascular smooth muscle cell expressing moderate GPR55 immunoreactivity. **(D, H)** Merged images (orange). Scale bar = 50 μm.

G protein-coupled receptor 55 immunoreactivity was expressed by the cytoplasm of synoviocytes and endothelial cells. In particular, faint-to-moderate GPR55-IR was mainly expressed by vimentin immunoreactive FLS showing elongated processes (Figures 7A–D). Only a few IBA1 immunoreactive MLS showed faint-to-moderate GPR55 (Figures 7E–H). Vascular endothelial cells and smooth muscle cells showed moderate GPR55-IR, respectively (Figures 7A–H). In the subintima, bright GPR55-IR was expressed by unidentified perivascular round-shaped cells (likely lymphocytes) which did not have IBA1-IR (Supplementary Figure 3).

### PPARα distribution and expression analysis

**Figure 8:**
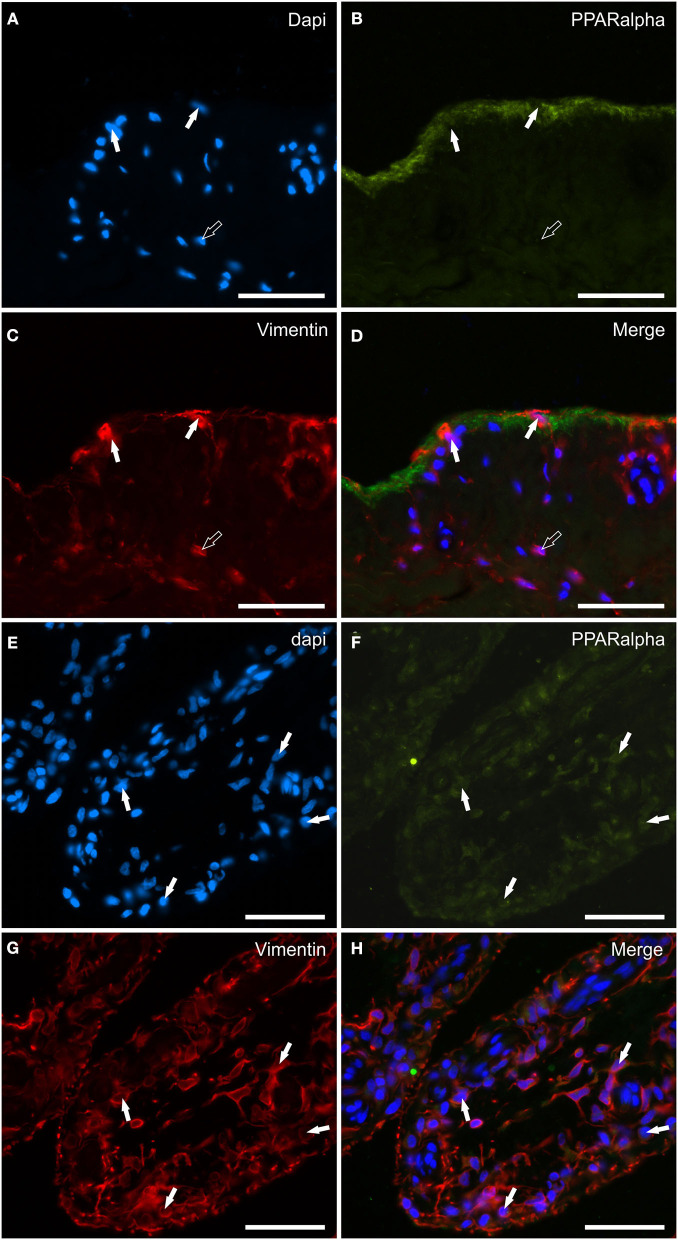
Photomicrographs of the cryosections of the synovial membrane of a horse metacarpophalangeal joint showing peroxisome proliferator-activated receptor alpha (PPARα) immunoreactivity in synoviocytes **(A–H)**. **(A–C)** The white arrows indicate the DAPI (Blue) labeled nuclei of synoviocytes brightly immunolabelled with the anti-vimentin (Red) antibody which expressed faint-to-moderate PPARα (Green) immunoreactivity. It is possible to see the indistinct PPARα immunostaining of the upper portions of the cells lining the joint cavity. **(E–H)** The figures show the longitudinal sections of two villi of the synovial membrane in which the arrows indicate the DAPI (Blue) labeled nuclei **(E)** of the cells, likely fibroblast-like synoviocytes and fibroblasts, co-expressing faint-to-moderate PPARα-(Green) **(F)** and bright vimentin-(Red) **(G)** immunoreactivity. **(D, H)** Merged images (Orange). Scale bar = 50 μm.

Peroxisome proliferator-activated receptor alpha immunoreactivity was expressed by synoviocytes, blood vessels and fibroblasts. The pattern of weak-to-moderate PPARα immunoreactivity was unusual as it appeared to be continuous, granular and indistinct immulolabeling of the cytoplasm of the upper portions/processes of the cellular elements facing the joint lumen (Figures 8A–D). The co-localization with the anti-vimentin antibody showed that the PPARα immunoreactive synoviocytes were most likely FLS (Figures 8E–H). No IBA1 immunoreactive synovial cells showed PPARα-IR (data not shown). Endothelial cells, as well as the smooth muscle cells of the blood vessels showed moderate cytoplasmic PPARα-IR; however, the PPARα-IR was more appreciable in large vessels (data not shown).

**Figure 9:**
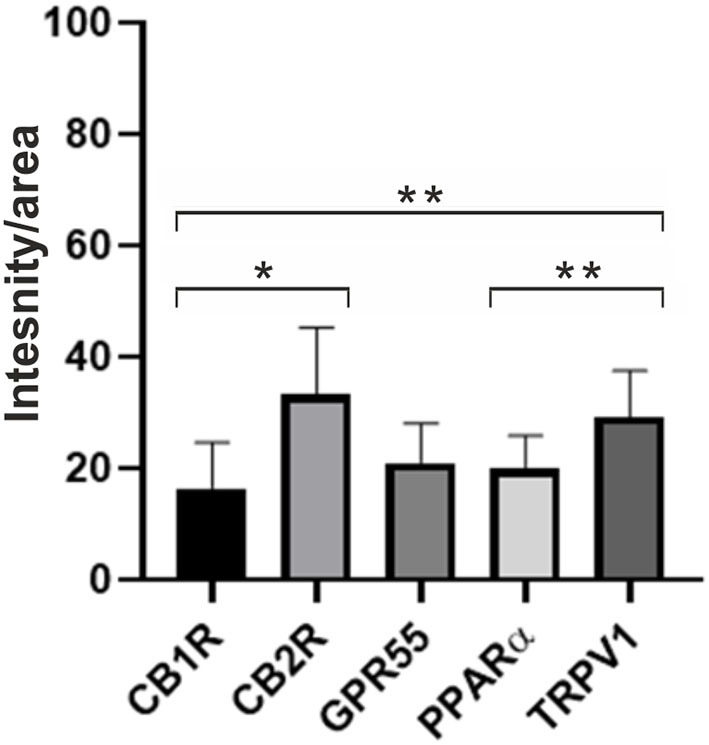
Quantification of the intensity of the expression of CB1R, CB2R, GPR55, PPARα, and TRPV1 in the synovial membrane of metacarpophalangeal joints of 12 horses. Data are represented as Mean ± SD and were analyzed using One-way ANOVA multiple comparisons test. *P < 0.05 and **P < 0.01.

Figure 9 shows the quantification of the intensity of the expression of CB1R, CB2R, GPR55, PPARα, and TRPV1 in the synovial membrane of the equine metacarpophalangeal joints.

**Figure 10:**
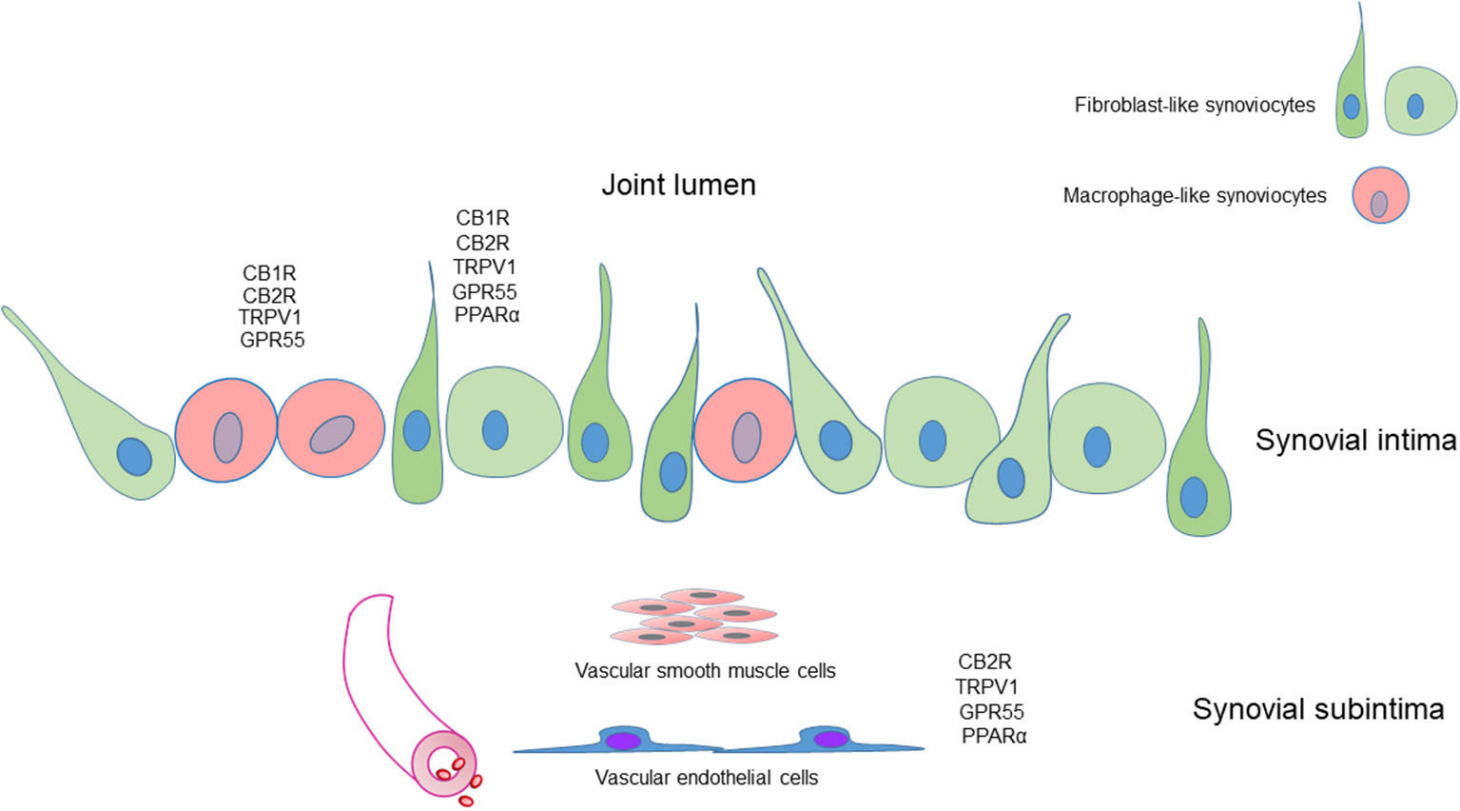
Graphical representation of the distribution of the cannabinoid receptors 1 (CB1R) and 2 (CB2R) and the cannabinoid-related receptors transient receptor potential vanilloid 1 (TRPV1), G protein-coupled receptor 55 (GPR55) and nuclear peroxisome proliferator-activated receptor alpha (PPARα) in the different cellular elements of the synovial membrane of the equine metacarpophalangeal joint. Fibroblast-like synoviocytes (FLS), identified with an anti-vimentin antibody, expressed CB1R, CB2R, TRPV1, GPR55, and PPARα immunoreactivity. Macrophage-like synoviocytes (MLS), identified with an anti-IBA1 antibody, expressed CB1R, CB2R, TRPV1, and GPR55 immunoreactivity.

Figure 10 shows the graphical representation of the distribution of the CB1R, CB2R, TPRV1, GPR55, and PPARα in the different cellular elements of the equine metacarpophalangeal synovial membrane.

## Discussion

Arthropathies can be a significant source of pain in horses, and finding new therapeutic treatments to alleviate the pain is of paramount importance (63). It is known that cannabis-based drugs have therapeutic potential in inflammatory diseases, including OA and rheumatoid arthritis (RA), as demonstrated by pre-clinical and clinical studies in animals and humans (28, 64). Interest in this type of molecule in horses has also recently been evidenced by a prospective, randomized, controlled study which attempted to determine the plasma pharmacokinetics, short-term safety, and synovial fluid levels of CBD following oral administration in horses (65).

Therefore, the localization of CB1R, CB2R, TRPV1, GPR55, and PPARα in the synovial FLS and MLS of the metacarpophalangeal joint of the horse is an encouraging finding.

Fibroblast-like synoviocytes are highly specialized mesenchymal cells found in the intimal lining layer of the synovium of diarthrodial joints. In a healthy joint, the FLS form a thin porous barrier at the interface between the sublining and the synovial fluid space (66). Fibroblast-like synoviocytes are pivotal cells in both joint maintenance and integrity, and in the inflammatory response/pathogenesis of arthritis (10, 67). The role of the FLS has also been highlighted in the pathogenesis of RA (15, 29, 68, 69). It has been recognized that, even in horses, FLS participate in the pathogenesis of joint disease by producing proinflammatory cytokines and cartilage-degrading mediators (70, 71). In horses with naturally occurring and experimentally induced OA and septic arthritis, increased levels of inflammatory components, such as leukocytes, interleukin (IL)-1β, IL-6, tumor necrosis factor α (TNF-α), and matrix metalloproteinases, has been demonstrated (72, 73). In RA, it has been shown that FLS become active upon stimulation by inflammatory cytokines released by macrophage-like synoviocytes (and T-lymphocytes) and secrete matrix metalloproteases (MMP), causing joint destruction (69).

Macrophages derive from two main cellular lineages; one lineage arises from bone-marrow-derived monocytes and the other is derived from cells which disperse into the tissues during embryonic development (23). The tissue-resident macrophages have distinctive gene-expression profiles which depend on the particular tissue in which they reside (25).

The three joint macrophage populations, i.e., the lining MLS, the sublining macrophages and the interstitial macrophages, differ in their origins and functions (74). In the healthy synovium, macrophages are predominantly monocyte-independent (20, 24, 74). The proliferation of macrophages harbored in the sublining connective tissue gives rise to both the MLS and the interstitial macrophages (24). In both mice and humans, lining MLS seem to be highly phagocytic and anti-inflammatory (74). In joint inflammation, the *synovium* also contains macrophages originating from recruited monocytes which produce pro-inflammatory cytokines and release molecules with the possibility of attracting lymphocytes which additionally propagate inflammation.

To add to the complexity, macrophages exist as various subsets, some of which are pro-inflammatory (M1) whereas others are anti-inflammatory and favor tissue repair (M2) (75, 76). Undoubtedly, in synovial inflammation and arthritis, monocytes and macrophages play a central role, promoting the onset and the progression of joint inflammation (74). In a recent study regarding the horse synovial membrane, M1 and M2 macrophages were characterized in normal and inflammed joints (26). It appears evident that, given the central role of macrophages in OA, a clinical approach targeting activated macrophages at an earlier stage of OA may serve to inhibit or slow the progression of disease (77).

In the current study, all the macrophage populations expressed IBA1-IR; in addition, also MLS, and sublining and interstitial macrophages expressed vimentin-IR with its stronger immunolabeling expressed by the MLS. Vimentin, which is the main intermediate filament protein in mesenchymal cells (such as epithelial cells and fibroblasts), has already been observed in rat (78) and human FLS (79). However, it has been reported that vimentin could also be expressed in the mononuclear phagocyte system (80); in particular, vimentin manifests enhanced fluorescence in activated macrophages (81). In the current study, only MLS showed bright vimentin-IR, evidence which suggested an activated state of the lining macrophages.

### Cnr1, Cnr2, GPR55, TRPV1, and PPARA gene expression in synoviocytes

To date, the gene expression has been reported in the equine synovial membrane only for *TRPV1* (4). The present study confirmed the expression of *TRPV1* and also demonstrated the expression of *Cnr1, Cnr2, GPR55*, and *PPARA*, according to the Authors' protein data. However, *Cnr1* were not expressed in all the horses.

### CB1R, CB2R, GPR55, TRPV1, and PPARα immunoreactivity in synoviocytes

Cannabinoid receptor 1, which is usually expressed by the neurons, also in horses (54, 82), has been identified in human and mouse synoviocytes (28, 31, 40). Cannabinoid receptor 1 has also been identified in synoviocytes of the horse (44) in which it was co-expressed with CB2R; the Authors were not able to identify the synovial cell types expressing CB1R-IR. Comparing the results of the current study with those described by Miagkoff et al. (43), some differences should be noted. The first difference is related to the notable expression of CB1R-IR in synoviocytes (greater when compared to CB2R-IR) noted by Miagkoff et al. (44). In the present study, the intensity of CB1R-IR was much lower than that of CB2R-IR; this evidence was also supported by the quantitative data of the mRNA *Cnr1* (although not necessary, a correlation between mRNA and protein expression exists). The difference between the results of the present study and that of Miagkoff et al. (44) did not lie in the use of different anti-cannabinoid receptor antibodies since the same anti-CB1R and -CB2R antibodies were used for both the studies. Instead, a plausible reason for this discrepancy in the results could be the use of sections of paraffin-embedded tissues which may often create a background in immunofluorescence reactions. Unlike what was observed in the Miagkoff et al. study (44), no CB1R-nuclear immunolabeling was observed in the current study. To avoid any tissue background, which might be an interference in the reading of weak receptor immunostaining, cryosections of the synovial membrane were used in the present study.

Cannabinoid receptor 2 is mainly expressed by the immune cells (53), and its activation is usually associated with a decrease in both immune cell function and cytokine release (83). Cannabinoid receptor 2 has been identified in human, mouse, rat, and horse synoviocytes (41–43).

Richardson et al. (28) identified CB2R (RNA and protein) in the FLS of healthy human patients, and patients with OA and AR. It has been shown that, in mouse and human joints, CB2R expression is up-regulated by proinflammatory mediators and injuries, and that its activation plays a key role in regulating inflammatory signaling in macrophages and FLS and suppresses the production of proinflammatory cytokines (42, 45, 84).

In the current study, CB2R-IR was brightly expressed in both FLS and MLS, suggesting a functional role of the endocannabinoid receptor system in horse joints. The evidence that targeting the CB2R in murine MLS and human FLS may be responsible for potent anti-inflammatory effects (45) could allow cautious speculation that the horse intra-articular ECS could be a promising therapeutic target for blocking pathological inflammation.

The TRP vanilloid 1 (TRPV1) ion channel is usually expressed by nociceptors of mammals (57, 85), including horses (53). However, TRPV1 is also expressed in various non-neuronal tissues, such as rat (41) and human (46) synoviocytes. Cells in synovial compartments can be exposed to low pH conditions after inflammation, infection, or injury. An acid sensing receptor (TRPV1) has been identified on synovial cells which are responsive to a low pH (pH 5.5–7.0) (46); TRPV1 is also activated by heat (>43°C) and capsaicin (86). In joint inflammation, the synovial compartments can also be exposed to thermal (>43°C), chemical, and osmotic modifications which can activate the TRPV1 membrane sensors which respond by activating calcium and sodium fluxes.

A number of studies have indicated that the TRPV1, which seems to mediate the calcium dependent proliferative and secretory responses of the synoviocytes in the event of joint inflammation, might be a possible and valuable target for treating joint diseases (46, 87), even in the horse (4). It has been shown that the TRP channels are functionally expressed in human synoviocytes and may play a critical role in adaptive or pathological changes in articular surfaces during arthritic inflammation, in particular in the response of the synoviocytes to the inflammatory mediator TNF-α (46). This evidence seems to have some therapeutic relevance, given that an *in vitro* study showed that the synovial cells from arthritic animals spontaneously produced large amounts of TNF-α (88).

The finding of TRPV1-IR in the FLS of the horse is consistent with those obtained in humans (46, 89), rats (41), and mice (31). The evidence of TRPV1-IR in MLS (and sublining macrophages) of the horse is also consistent with what has already been observed in human MLS (89). Gene expression and immunohistochemical data strictly correlate and integrate with the recent observations of Braucke et al. (4) who identified and quantified the TRPV1 mRNA and the TRPV1 protein level in the metacarpo/metatarsophalangeal joints of the horse, and observed a higher expression of TRPV1 in samples from joints with pathology.

It has been shown that TRPV1 inhibits M1 macrophage polarization in the synovium and attenuates the progression of OA in a rat model of OA (90). In addition, Engler et al. (91) showed that stimulation of the cultured synovial fibroblasts of OA and RA in human patients with capsaicin (TRPV1 agonist) led to the increased expression of IL-6 mRNA and IL-6 protein, and that IL-6 protein expression could be antagonized with capsazepine (a TRPV1 antagonist). Therefore, TRPV1 may play a role in non-neuronal mechanisms which could modulate nociception in symptomatic OA and RA patients.

Vanilloid receptor 1 (VR1 or TRPV1) is desensitized by endovanilloids, endocannabinoids (anandamide), endocannabinoid-like molecules (92, 93) and phytocannabinoids, such as CBD (38, 94) which shows anti-nociceptive, analgesic, and anti-inflammatory effects (35, 95). The importance of the endocannabinoid signaling acting on TRPV1 has been highlighted by different OA studies in which it has been shown that synovial fibroblasts express several receptors involved in endocannabinoid action, and that endocannabinoid anandamide (AEA) reduces IL-6, IL-8, and TNF-α production by mixed synoviocytes (31).

Studies involving phytocannabinnoids showed that CBD, targeting synovial fibroblasts under inflammatory conditions, demonstrated anti-inflammatory effects on arthritis (96). Cannabidiol may exert its anti-inflammatory and protective effects *via* TRPV1 receptors, as shown in the *in vitro* LPS-stimulated murine macrophage cell line (97). In mice, it has been shown that synovial cells treated with CBD produced significantly less TNFα in culture and that CBD suppressed clinical signs of the disease without obvious side effects during chronic treatment (27). Since CBD binds to several other receptors (TRPA1, GPR55, PPAR gamma, serotonin receptors, etc.), its mode of action remains elusive. However, CBD reduces IL-6/IL-8/MMP-3 production of RA synovial fibroblasts (96).

Not only phytocannabinoids but also the synthetic cannabinoid WIN55,212-2 mesylate (WIN) demonstrated strong anti-inflammatory effects in monocytes and synovial fibroblasts *via* a TRPV1 (and TRPA1) dependent pathway (12).

G protein-coupled receptor 55 (GPR55), which is considered to be the third cannabinoid receptor, has been identified in the sensory neurons of different species, including dogs, rats (57) and horses (53), and in canine inflammatory cells (58). In addition, GPR55 has also been localized in human chondrocytes (48), osteoclasts and osteoblasts (47), and seems to be associated with bone remodeling and vascular homeostasis (98). To the best of the Authors' knowledge, no data are available regarding the expression of GPR55 in synoviocytes and subintimal synovial cells. The expression of GPR55-IR has recently been shown in the macrophages harbored within the horse dorsal root ganglia (53). A study on rodents has shown that the peripheral activation of GPR55 can reduce mechanosensitivity in the event of joint inflammation (99). However, it is not clear whether this effect was exerted only at the level of the peripheral and central nervous system or also locally, at the level of the synovial cells.

In the present study, GPR55-IR has been demonstrated in both FLS, MLS, subintimal macrophages, and unidentified inflammatory/immunitary cells, suggesting an active role of the receptor in synovial membrane homeostasis and immunity. Cannabidiol, which acts as a GPR55 antagonist, should be able to reduce the migration of macrophages, as shown in mice (100).

Peroxisome proliferator-activated receptor alpha seems to have a role in sensory modulation due to its expression in the sensory neurons of animals, including horses (54).

Peroxisome proliferator-activated receptor alpha can be expressed by different cells of innate immunity, including monocytes and macrophages (101). A number of studies have documented the anti-inflammatory consequences of PPARα activation in human and murine macrophages (102, 103). Ligands of PPAR-α have been shown to regulate inflammatory responses (104) so much so that, in PPAR-α deficient mice, abnormally prolonged responses to different inflammatory stimuli have been noted (105). The endogenous and exogenous PPAR-alpha ligands reduce the degree of macrophage inflammation caused by LPS/IFN-gamma stimulation (106).

There are studies indicating that PPARα agonists may exert beneficial effects on OA due to their anti-inflammatory effects (49). Fenofibrate, a PPAR-alpha ligand, has been shown to inhibit the development of arthritis in a rat model of human RA by reducing cytokine production (IL-6, IL-8 and granulocyte monocyte colony-stimulating factor) from FLS (107).

There is extensive documentation regarding the anti-inflammatory, analgesic, immunomodulatory and neuroprotective effects of the endocannabinoid-like lipid mediator PEA, also for joint health and pain modulation (108, 109). Palmitoylethanolamide exerts its analgesic and anti-inflammatory effects primarily by activating the PPAR-α; however, binding to PPAR-α, PEA triggers TRPV1 channel activation, providing another mode of action in which PEA interacts with the endocannabinoid and endovanilloid systems (110).

### CB1R, CB2R, GPR55, TRPV1, and PPARα immunoreactivity in synovial blood vessels

Endothelial cells and smooth muscle cells were positive for analyzed markers. However, since among cells surrounding endothelial cells there are not only smooth muscle cells but also pericytes as well as adventitial cells (111, 112) and no specific markers for these cellular cytotypes have been used, it cannot be excluded that cannabinoid and cannabinoid-related receptors may also be expressed by other vascular cells.

The principal functions of the endothelium are to promote smooth muscle cell relaxation and arterial dilation, control vascular permeability, exert an antithrombotic effect, and regulate angiogenesis (113).

The angiogenesis may exacerbate OA pain, and the upregulated angiogenic factors and the molecules produced by vascular cells may also stimulate nerve growth (7, 114). In addition to the role they play regarding pain, neuropeptides released by stimulated nerve endings are involved in vasodilation, inflammation (by producing proinflammatory cytokines and by activating inflammatory infiltrating cells), and synoviocyte proliferation and activation (115, 116). During joint diseases, the proliferation of endothelial cells and their morphological differentiation to form tubes accompanies extracellular matrix degradation which facilitates the tissutal invasion of inflammatory cells and is perpetuated by various mediators (2, 116–118). Therefore, angiogenesis and matrix degradation may be interesting/key targets to counteract the progression and chronicity of joint inflammation and degeneration.

Cannabinoids are hypotensive and vasodilator molecules which can exert their effects by acting on the vascular smooth muscle cells and/or endothelial cells (119).

Cannabinoid receptor 1 has been observed in both vascular cellular elements (120) in which it exerts vasodilatatory effects. However, in the present study, any CB1R-IR was observed in the capillaries or larger blood vessels of the horse *synovium*; this finding was also in contrast to the data published by Miagkoff et al. (44).

In the present study, the expression of CB2R-IR by vascular endothelial and smooth muscle cells was described. The Expression of CB2R-IR has previously been observed in the vascular endothelial cells of humans and animals (57, 121, 122), including horses (44).

The expression of CB2R-IR in blood vessels may functionally be in relation to the data observed in normal joint of rats in which it has been shown that the CB2R agonist JWH133 caused hyperemia *via* a CB2R and TRPV1 mechanism, and that, during acute and chronic inflammation, this vasodilatatory response was significantly attenuated (122). Rajesh et al. (123), by investigating the effect of CB2R receptor agonists on TNFα-induced proliferation, migration and signal transduction in the smooth muscle cells of human coronary arteries, observed that CB2R agonists decreased vascular smooth muscle proliferation and migration. Although for the most part, hypothetically and totally not demonstrated, the Authors cannot exclude that CB2R agonists might also reduce the angiogenesis and inflammation in an inflamed horse joint.

In the current study, TRPV1-IR was observed in both the endothelial and smooth muscle cells of the synovial blood vessels, a finding consistent with that obtained in humans and other animals (124–127).

The notable anti-angiogenic activities of cannabinoid compounds, which have mainly been tested in tumor experiments, are carried out directly, inhibiting vascular endothelial cell migration and survival, and decreasing the expression of proangiogenic factors (35, 128). It has been shown that CBD may inhibit angiogenesis by the down-modulation of several angiogenesis-related molecules (117). Cannabinoids may act on different receptors to obtain their effect; however, the expression of TRPV1-IR in the endothelial cells of the horse synovial membrane is relevant as it is known that TRPV1 promotes endothelial cell proliferation and network-formation by means of the cellular uptake of the endocannabinoid anandamide (127). Therefore, CBD, which stimulates and desensitizes TRPV1, may potentially contrast angiogenesis in horse joint inflammation.

The endothelium exerts a profound relaxing effect on the underlying smooth muscle cells; nitric oxide (NO) is a well-characterized vasoactive substance produced by the endothelium which diffuses to and relaxes the smooth muscle, causing arterial dilation (129). It has been shown that CBD causes vasorelaxation of the human mesenteric arteries *via* activation of the CB1 and TRPV1 channels, and it is endothelium- and nitric oxide-dependent (129).

G protein-coupled receptor 55 was observed in the blood vessels of the horse joint, a finding which is consistent with that of Xu et al. (130) who identified GPR55 in the endothelium of human and mouse aortas, and of Daly et al. (131) who located GPR55 in the endothelium of mouse blood vessels. The first evidence of the functional role of GPR55 was obtained in the vascular system in which it was shown to regulate systemic vascular resistance and angiogenesis (98, 132). Scientific evidence indicates that the agonists of GPR55 can elicit either vasoconstriction or vasorelaxation (133). Recent studies involving humans have indicated that L-α-lysophosphatidylinositol, a GPR55 agonist, induced endothelium-dependent vasorelaxation in the pulmonary arteries (134) and mediated ovarian carcinoma cell-induced angiogenesis (135). Due to the antagonist effect of CBD on GPR55, it is reasonable to consider, although in a purely speculative way, that CBD might reduce angiogenesis and vasorelaxation of the blood vessels of the horse joint *via* GPR55.

Also the expression of PPARα-IR was observed in the endothelial cells of the horse joint, as has already been described in the blood vessels of the cervical DRG (53). The anti-proliferative and anti-angiogenic properties of PPARα in endothelial cells have been demonstrated in a variety of *in vitro* and *in vivo* models (104). Taken together, these findings lead us to hypothesize that the analgesic and anti-inflammatory properties of this receptor, as previously described in other species, are also present in the horse (38, 136).

The present study demonstrated that, in the equine synovial membrane in healthy joints, the mRNA of *Cnr1, Cnr2, TRPV1, GPR55*, and *PPARA* was present, according to the protein results. Moreover, the mRNA results of *TRPV1* were consistent with a previous study regarding equine articular tissue (4). To the best of the Authors' knowledge, no data have been reported on equine articular tissue regarding the expression of the other receptors described in this paper.

### Limitation

There are some limitations which should be taken into consideration when interpreting the results of this study. It cannot be ruled out that some factors could potentially alter the CB1R, CB2R, TRPV1, GPR55, and PPARα expression in tissues, such as the unknown underlying pathological conditions of the horses in the study or the medications received. In addition, the limited number of horses considered in the current study, the reduced representation of male to female horses, as well as adult and young horses, represent another limitation of the study.

## Conclusion

The present study was the first study to demonstrate the mRNA presence and the protein cellular distribution of the cannabinoid receptors (CB1 and CB2) and three cannabinoid-related receptors (TRPV1, GPR55, and PPARα) in the horse synovial tissues of the metacarpophalangeal joint of the horse. Cannabinoid receptor 1 was identified in FLS and MLS, although it was not expressed in all the horses. Cannabinoid receptor 2, TRPV1 and GPR55 were identified in FLS, MLS, and blood vessels, while PPARα-IR was identified in FLS and blood vessels. Due to their cellular localization, these receptors may be the target of many drugs (endocannabinoids and endocannabinoid-related molecules, non-psychoactive phytocannabinoids, synthetic cannabinoids and several agonist and antagonist drugs) which could potentially be utilized to improve inflammation and pain in horses with joint diseases. These results should hopefully encourage the development of new molecular and preclinical studies supporting the use of molecules already tested and used in humans and animals which could potentially reduce the joint inflammation in horses with joint diseases. Comparison of the data of the current study with the data obtained from the synovial tissues of horses with metacarpophalangeal joint disease could be of interest to verify whether mRNA of *Cnr1, Cnr2, TRPV1, GPR55*, and *PPARA*, and the immunoreactivity for the same receptors are up- or down-regulated during joint disease.

## Data availability statement

The raw data supporting the conclusions of this article will be made available by the authors, without undue reservation.

## Ethics statement

Ethical review and approval was not required for the animal study because the metacarpophalangeal joints of horses slaughtered for consumption were collected post-mortem. According to Directive 2010/63/EU of the European Parliament and of the Council of 22 September 2010 regarding the protection of animals used for scientific purposes, the Italian legislation (D. Lgs. no. 26/2014) does not require any approval by competent authorities or ethics committees because this study did not influence any therapeutic decisions.

## Author contributions

RC, RZC, RR, and AG contributed to the study design. The mRNA analysis was carried out by AZ and MF. The immunohistochemical experiments were carried out by RZC, MDS, and GS. Acquisition of data and drafting of the manuscript was done by RC. All authors interpreted the data. All authors contributed to the study execution and approved the final manuscript.
