# Supplementary material for: Expression of cannabinoid (CB1 and CB2) and cannabinoid-related receptors (TRPV1, GPR55, and PPARα) in the synovial membrane of the horse metacarpophalangeal joint
Source: Front Vet Sci. 2023 Mar 3;10:1045030. doi: 10.3389/fvets.2023.1045030 (PMC10020506; doi:10.3389/fvets.2023.1045030)
Supplement: Supplementary file 1 [file Data_Sheet_1.PDF]

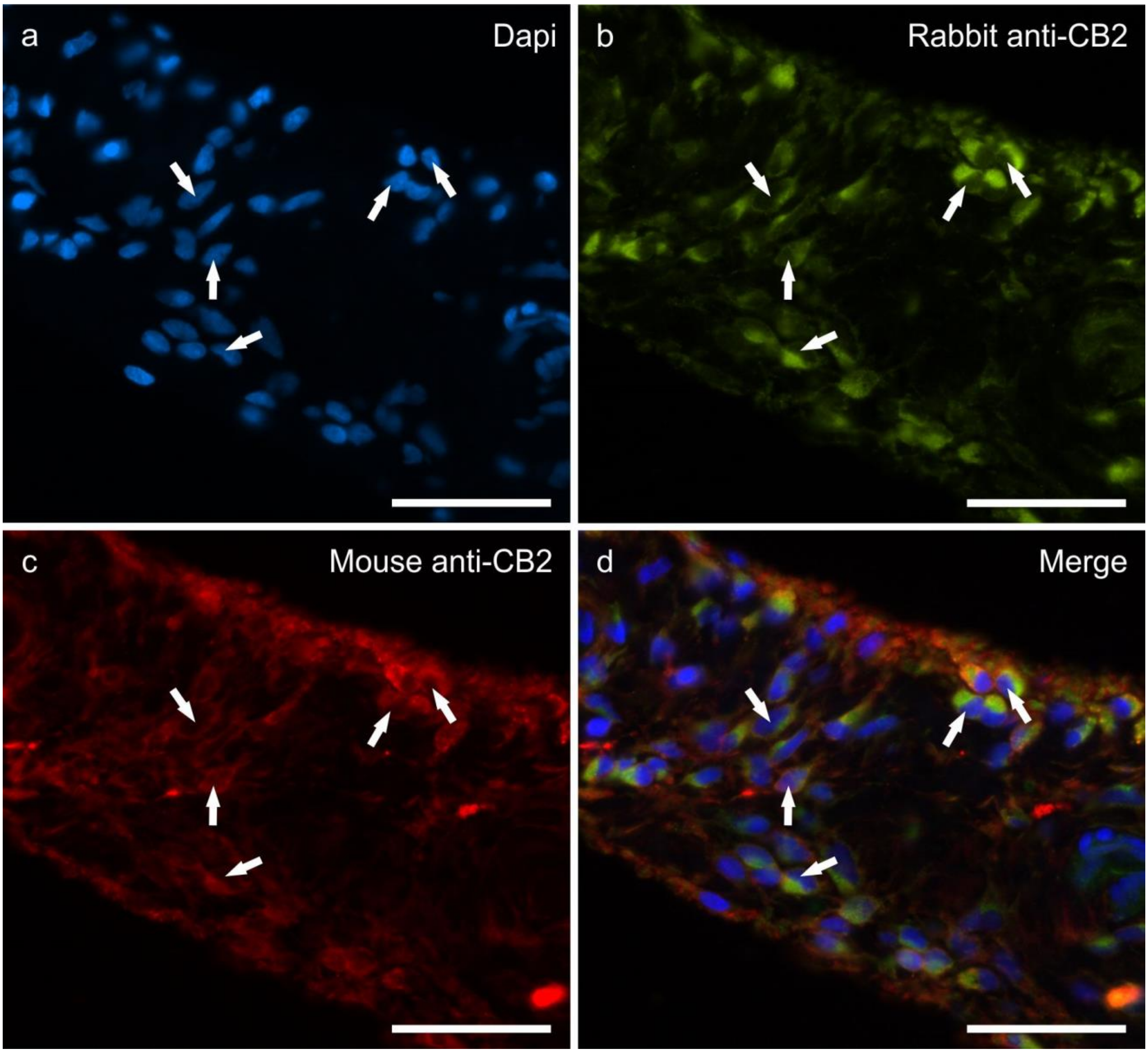

Figure S1. a-d) Photomicrographs of a cryosection of a villus of the synovial membrane of the horse metacarpophalangeal joint. Two different antibodies against cannabinoid receptor 2 (CB2), raised in rabbit (b) and mice (c), were used to identify the synoviocytes. The arrows indicate some Dapi labelled nuclei of cells in which the two antibodies were co-localised.

Bar: a-d = 50 µm
