# Supplementary material for: Expression of cannabinoid (CB1 and CB2) and cannabinoid-related receptors (TRPV1, GPR55, and PPARα) in the synovial membrane of the horse metacarpophalangeal joint
Source: Front Vet Sci. 2023 Mar 3;10:1045030. doi: 10.3389/fvets.2023.1045030 (PMC10020506; doi:10.3389/fvets.2023.1045030)
Supplement: Supplementary file 2 [file Data_Sheet_2.PDF]

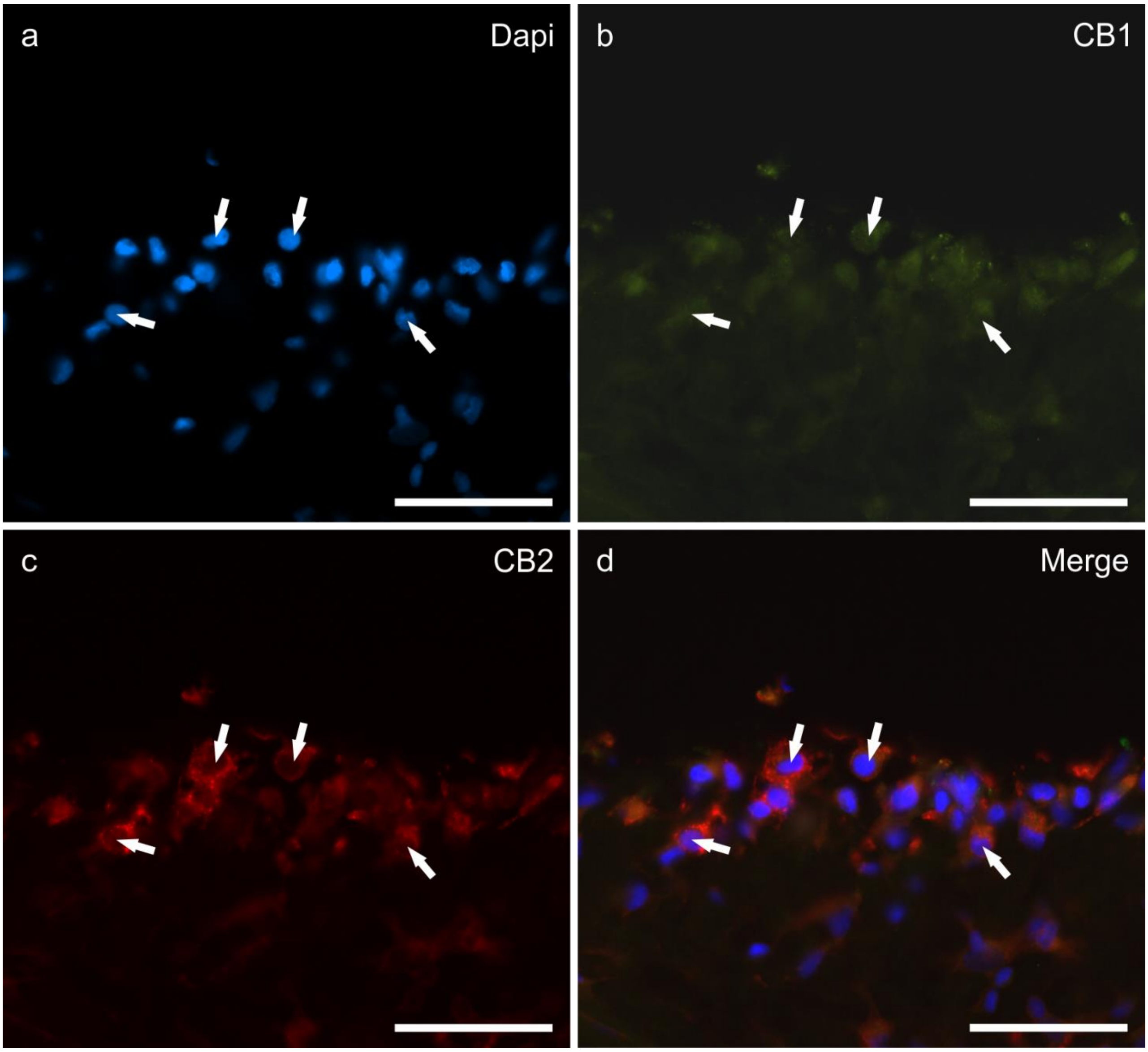

Figure S2. a-d) Photomicrographs of a cryosection of the synovial membrane of the horse metacarpophalangeal joint. The white arrows indicate the Dapi labelled nuclei of some synoviocytes which co-expressed weak immunoreactivity for cannabinoid receptor 1 (CB1) (b) and bright immunoreactivity for cannabinoid receptor 2 (CB2) (c).

Bar: a-d = 50 µm
