# Supplementary material for: Expression of cannabinoid (CB1 and CB2) and cannabinoid-related receptors (TRPV1, GPR55, and PPARα) in the synovial membrane of the horse metacarpophalangeal joint
Source: Front Vet Sci. 2023 Mar 3;10:1045030. doi: 10.3389/fvets.2023.1045030 (PMC10020506; doi:10.3389/fvets.2023.1045030)
Supplement: Supplementary file 3 [file Data_Sheet_3.PDF]

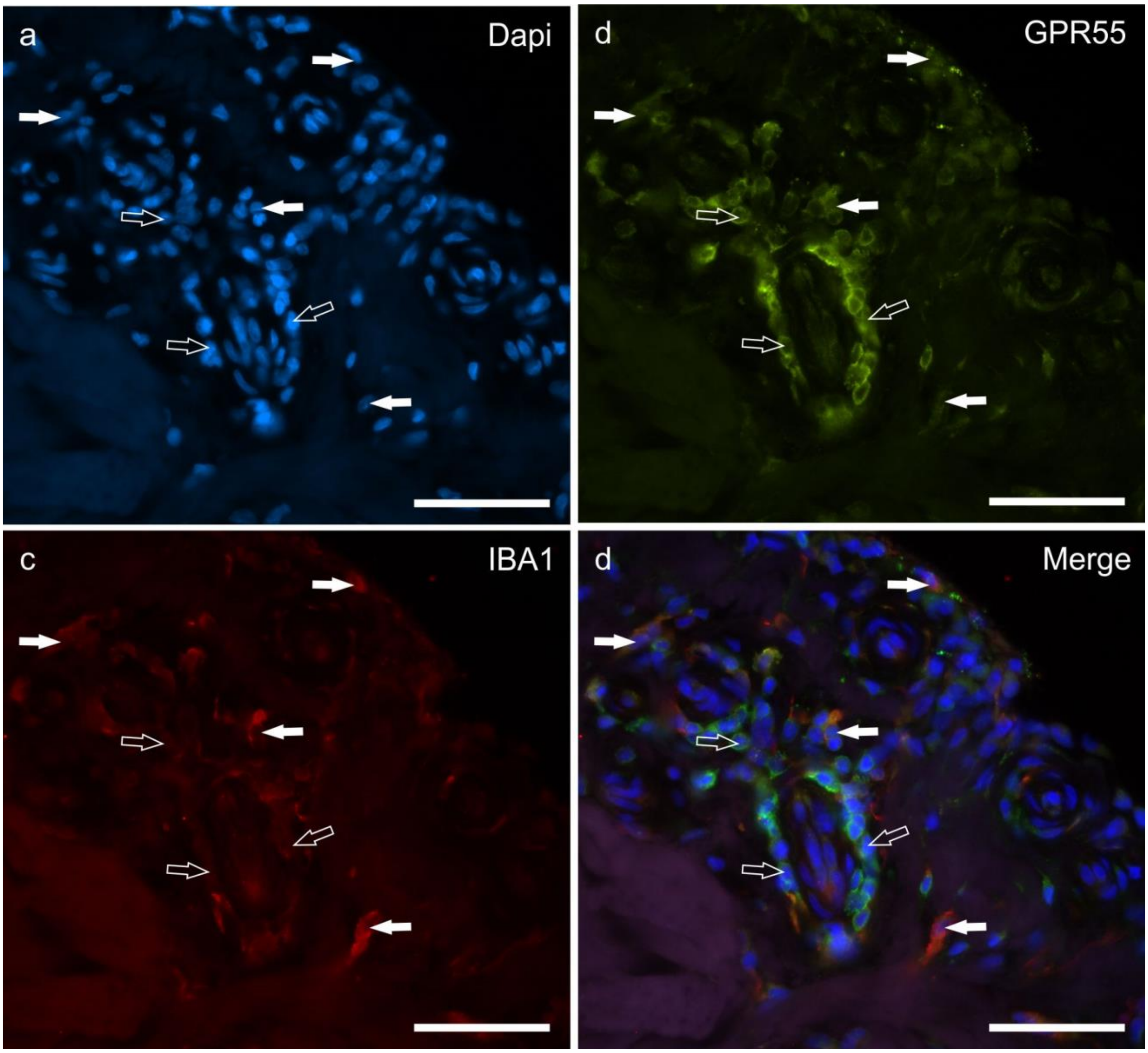

Figure S3. a-d) Photomicrographs of a cryosection of the synovial membrane of the horse metacarpophalangeal joint. The white arrows indicate the Dapi labelled nuclei of some interstitial macrophages which co-expressed immunoreactivity for G protein-coupled receptor 55 (GPR55) (b) and IBA1 (c). The open arrows indicate the Dapi labelled nuclei of some perivascular IBA1 negative cells, which expressed bright GPR55 immunoreactivity.

Bar: a-d = 50 µm
